# Prevalence of Thyroid Dysfunction in Patients With Chronic Spontaneous Urticaria Attending a Tertiary Care Urticaria Clinic: A Cross-Sectional Study

**DOI:** 10.7759/cureus.113619

**Published:** 2026-07-29

**Authors:** Varsha Srinivasan, Rajkumar Kannan, Sampath Vadivelu

**Affiliations:** 1 Medicine, Madras Medical College, Chennai, IND; 2 Dermatology, Venereology and Leprology, Madras Medical College, Chennai, IND

**Keywords:** associations of chronic urticaria, autoimmune thyroiditis, hypothyroidism, impact of correction of hypothyroidism, pattern of hive, tsh levels

## Abstract

Background and objectives

Existing studies have primarily focused on autoimmune thyroid disorders in patients with chronic spontaneous urticaria (CSU); fewer studies have assessed thyroid function using routine thyroid function tests alone. The purpose of this study was to estimate the prevalence and pattern of common thyroid disorders among CSU patients at a tertiary care center.

Methods

A descriptive cross-sectional study was conducted among 123 patients aged above 18 years with CSU visiting the Urticaria OPD at Rajiv Gandhi Government General Hospital. After obtaining written informed consent, patients were assessed using a validated, interviewer-administered questionnaire and examined clinically. All patients underwent serum thyroid function testing, including free T3, free T4, and thyroid-stimulating hormone estimation by chemiluminescence Immunoassay. Data were entered into Microsoft Excel and analyzed using IBM SPSS Statistics for Windows, Version 26 (Released 2018; IBM Corp., Armonk, New York, United States) to determine the prevalence and pattern of thyroid disorders among CSU patients. Chi-square, Fisher's exact test, Mann-Whitney U test, and odds ratios with 95% confidence intervals were used for inferential analysis.

Results

The prevalence of thyroid abnormalities among patients with CSU was 29 of 123 patients (23.5%; 95% CI: 16.9-31.8%). Hypothyroidism was observed in 28 (22.8%) patients and constituted the majority of abnormalities, while hyperthyroidism was observed in one patient (0.8%). Thyroid dysfunction was observed in 16 (28.0%) patients aged <45 years and 13 (19.6%) patients aged ≥45 years (p=0.339). Female patients showed higher rates of thyroid dysfunction (27.1%) compared to male patients (15.8%), though this difference was not statistically significant (p=0.174).

Conclusion

The relatively high prevalence of thyroid dysfunction observed in this cohort suggests that thyroid function assessment may be considered in selected CSU patients, particularly where clinical suspicion exists.

## Introduction

Urticaria is mast cell-driven sudden appearance of itchy, erythematous wheals of variable size involving the epidermis and dermis with or without angioedema. Based on the duration of the symptoms, it is divided into acute urticaria (less than six weeks) and chronic urticaria (six weeks and more) [1]. Chronic urticaria is further classified as inducible and spontaneous based on the presence or absence of environmental triggers. The point prevalence of chronic spontaneous urticaria (CSU) is estimated to be 0.1%-1% [2]. Urticarial vasculitis, which may be accompanied by hypocomplementemia and systemic symptoms, is a crucial differential diagnosis of chronic urticaria. A biopsy should be done to rule out this entity if urticarial lesions last longer than 24 hours and also if the lesions are painful.

It is commonly acknowledged that patients with CSU frequently have additional underlying diseases, such as autoimmune diseases. About 30% to 50% of spontaneous instances of CSU have been linked to autoimmune urticaria [3]. The most frequent autoimmune condition in CSU patients is thyroid dysfunction [3]. Antithyroid antibodies are present in 5% to 34% of CSU patients, and another 5% to 10% have thyroid illness that has been clinically or biochemically verified [3].

The pathophysiology that links CSU and thyroid dysfunction involves multiple immunological mechanisms. Thyroid-stimulating hormone (TSH) receptors are expressed not only in thyroid tissue but also on immune cells, and TSH may act as a cytokine that stimulates lymphocytes and dendritic cells to release IL-1, IL-2, IL-6, and IL-12. This enhances mast cell activation [4]. Moreover, antithyroid antibodies have been detected in up to 27% of CSU patients, suggesting a shared autoimmune link between CSU and thyroid disease [5].

As much of the existing literature has analyzed only the prevalence of autoimmune thyroid disorders in CSU, the relationship between other thyroid abnormalities and CSU that can be detected in routine thyroid function testing alone has not been well studied in this population. Given that routine thyroid function tests are more accessible across all levels of the public healthcare system in India, unlike autoantibody testing, it is important to evaluate the prevalence of such thyroid disorders in CSU patients and analyze their coexistence with CSU characteristics. Screening for thyroid function may be considered in all patients with CSU for early identification of patients who require treatment of underlying thyroid dysfunction or follow-up. The observed coexistence of CSU and thyroid dysfunction may explain why CSU improves in response to levothyroxine or other thyroid supplements [6-8]. 

Aims and objectives

Primary Objective

The primary objective was to estimate the prevalence of thyroid disorders in patients with CSU.

Secondary Objectives

The secondary objectives were to determine the most prevalent thyroid disorder in patients with CSU and to explore the association between thyroid dysfunction and clinical characteristics of CSU including age, sex, and cutaneous manifestations.

## Materials and methods

This cross-sectional study was conducted in the Urticaria Outpatient Department of Rajiv Gandhi Government General Hospital, Chennai over a period of 10 months from March 2023 to December 2023. The study included patients aged more than 18 years of both genders who were diagnosed with CSU and were willing to provide informed consent. Patients who had undergone total thyroidectomy or were already on thyroid hormone supplementation were excluded from the study. The diagnosis of CSU was made by a licensed dermatologist who is a subject expert, based on clinical history and examination findings.

Urticaria was defined as the presence of itchy, erythematous wheals of variable size involving the epidermis and dermis, with or without associated angioedema. Chronic urticaria was defined as the persistence of symptoms for more than six weeks.

The sample size was calculated assuming a prevalence of thyroid disorders among CSU patients to be 7.5%, based on previously published literature. With a 95% confidence interval (CI) and an absolute precision of 5%, the calculated sample size was 107. After accounting for a 10% non-response rate, the minimum sample size was calculated to be 117 patients. To minimize selection bias, all consecutive patients meeting the eligibility criteria during the study period were included, resulting in a final sample size of 123.

The study commenced after obtaining approval from the Institutional Ethics Committee of Madras Medical College, Chennai (EC Reg. No: CDSCO.ECR/270/Inst./TN/2013/RR-20; DHR EC Reg. No: EC/NEW/INST/2021/1618). An information sheet containing the title of the study, explanation of the study tools, voluntary nature of participation, and declaration of confidentiality was included with the consent form. Written informed consent was obtained in the regional language from all the study patients.

A structured questionnaire was developed by the investigator to collect data relevant to the study objectives under the supervision of the department faculty, who also reviewed it for content and face validity before data collection. Formal pilot testing was not conducted. It consisted of three sections: socio-demographic details, clinical history of urticaria, and findings from general and dermatological examination. The questionnaire was validated, with a Cronbach’s alpha of 0.8. It was administered in the local language, and all interviews were conducted by a single investigator to minimize inter-observer variability. Each interview took approximately 5-10 minutes. The structured questionnaire used in the study is provided in the appendices section. Inducible urticarias including dermographism, cholinergic urticaria, cold urticaria, and pressure urticaria, urticarial vasculitis, and drug-induced urticaria were excluded through detailed history taking and clinical examination using the questionnaire. 

Involvement of palms and soles, hair and nails, and laryngeal edema were specifically assessed as these regions can possibly affect quality of life for the patient and clinical management in chronic urticaria. Hair and nail involvement was defined as urticarial lesions affecting the hair bearing scalp or nail apparatus. Laryngeal edema was defined as reported episodes of throat tightening, hoarseness, or difficulty swallowing associated with urticarial episodes reported by the patients. Associated ENT concerns included symptoms related to the ear, nose, and throat reported during urticarial episodes. Associated dental concerns included oral mucosal involvement or perioral angioedema reported by patients.

After obtaining consent, all patients included in the study were subjected to a serum thyroid profile test, which estimated the serum free T3, free T4, and TSH by chemiluminescence immunoassay at the institutional laboratory. Blood samples were collected for testing the thyroid profile of patients after an overnight fast of at least 12 hours. If not fasting, patients were instructed to observe 12-hour fasting and return on a later date. Age-appropriate reference ranges were applied as follows: TSH 0.40-4.20 µIU/mL (21-54 years) and 0.50-8.90 µIU/mL (55-87 years); free T4 0.80-2.70 ng/dL (21-87 years); free T3 2.30-4.20 pg/mL (>18 years). Patients were classified as hypothyroid, hyperthyroid, or euthyroid based on TSH levels. Hypothyroidism was defined as TSH above the upper reference limit for age. Hyperthyroidism was defined as TSH below the lower reference limit for age. Patients with TSH within the age-appropriate reference range were classified as euthyroid. While TSH was the primary parameter used for thyroid status classification, FT3 and FT4 were measured concurrently to provide a comprehensive thyroid profile and to identify patients with discordant values. The investigations were done free of charge in laboratories in the institution, affiliated with the Department of Dermatology.

Data were entered into Microsoft Excel and analyzed using IBM SPSS Statistics for Windows, Version 26 (Released 2018; IBM Corp., Armonk, New York, United States). Normality of continuous variables was assessed using the Shapiro-Wilk test. All continuous variables were found to be non-normally distributed on the Shapiro-Wilk test and are reported as median with interquartile range (IQR). Categorical variables, including gender distribution, thyroid status, and anatomical distribution of urticarial lesions, are expressed as frequencies and percentages. The relationship between categorical variables was assessed using the chi-square test or Fisher's exact test where cell counts were less than five. The Mann-Whitney U test was used to compare continuous variables between two independent groups. Odds ratios with 95% CIs were calculated for relevant categorical associations. A p-value of <0.05 was considered statistically significant.

## Results

The responses and blood test results of 123 patients were analyzed using appropriate statistical tests.

A total of 123 CSU patients participated in the study. The median age of patients was 45 years (IQR: 34.5-52.0 years). The median duration of urticaria was 24 months (IQR: 12-60 months). The median duration of urticaria did not differ significantly between patients with thyroid dysfunction and euthyroid patients (Mann-Whitney U=1257, p=0.525, effect size r=0.08).

All 123 patients with CSU who were included in the study were subjected to serum thyroid profile analysis.

Out of 66 (53.6%) patients aged 45 years and above, 13 (19.6%) had thyroid abnormalities. Of those, 12 (18.1%) patients had hypothyroidism and one (1.5%) patient had hyperthyroidism. Among the 57 (46.3%) patients aged less than 45 years, 16 (28%) had hypothyroidism, while none had hyperthyroidism (Table 1). There were a total of 38 (30.9%) male CSU patients, of whom six patients (15.7%) had hypothyroidism. Out of the 85 (69.1%) female CSU patients, 23 (27%) had thyroid abnormalities (Table 1).

**Table 1:** Age-wise and sex-wise categorization of thyroid status of patients

Age Category	Thyroid Status			Total n (%)	p-value
	Euthyroid n (%)	Hyperthyroid n (%)	Hypothyroid n (%)		
45 years and above	53 (80.3)	1 (1.5)	12 (18.1)	66 (53.6)	p=0.339
< 45 years	41 (71.9)	0 (0.0)	16 (28.0)	57 (46.3)	
Male	32 (84.2)	0 (0.0)	6 (15.7)	38 (30.9)	p=0.174
Female	62 (72.9)	1 (1.2)	22 (25.8)	85 (69.1)	
Total	94 (76.4)	1 (0.8)	28 (22.7)	123 (100)	

**Figure 1:**
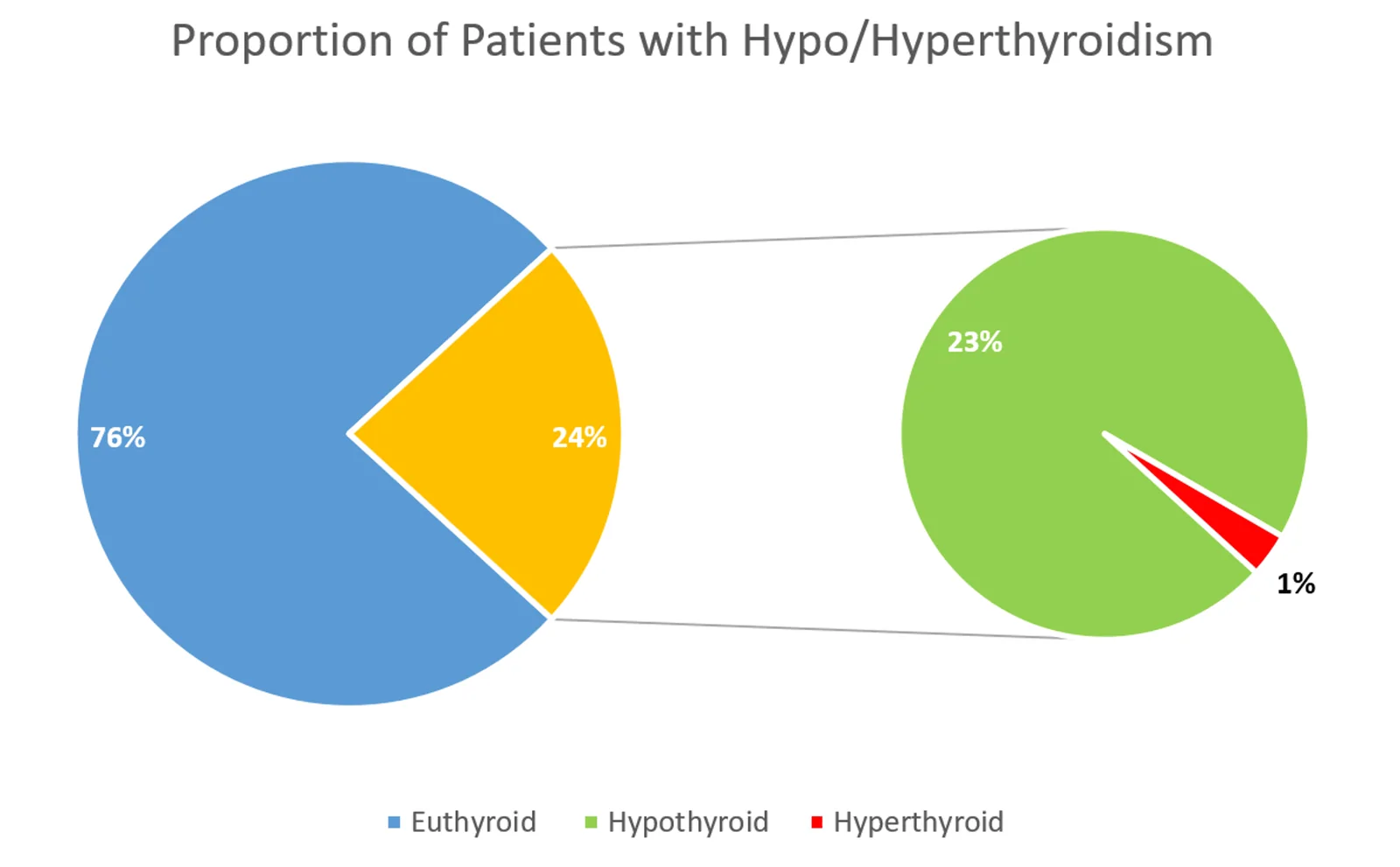
Proportion of chronic spontaneous urticaria patients with thyroid abnormalities

Among the 123 CSU patients tested, 29 (23.5%, 95% CI: 16.9%-31.8%) had thyroid abnormalities. Of these patients, 28 (22.8%, 95% CI: 15.6%-31%) had hypothyroidism, and one (0.8%, 95% CI: 0.02%-4.4%) had hyperthyroidism (Figure 1). Thyroid dysfunction was observed in 28.0% of patients aged <45 years and 19.6% of those aged ≥45 years, with no statistically significant difference (χ²=0.915, p=0.339; OR=1.504, 95% CI: 0.650-3.479). Female patients showed higher rates of thyroid dysfunction (27.1%) compared to male patients (15.8%), though this did not reach statistical significance (χ²=1.851, p=0.174; OR=0.505, 95% CI: 0.187-1.367).

The median values of the thyroid parameters fall within the normal range of the serum levels, with a relatively small IQR (Table 2).

**Table 2:** Thyroid profile of patients IQR: Interquartile range

Thyroid Parameters	Thyroid Profile of Patients		
	Range	Median	IQR
Thyroid-stimulating hormone (in µIU/mL)	0.25 – 30.1	2.97	1.77 – 4.30
Tetraiodothyronine (in ng/dL)	0.15 – 14.2	0.86	0.77 – 1.38
Triiodothyronine (in pg/mL)	0.63 – 13.1	2.92	2.26 – 3.20

**Figure 2:**
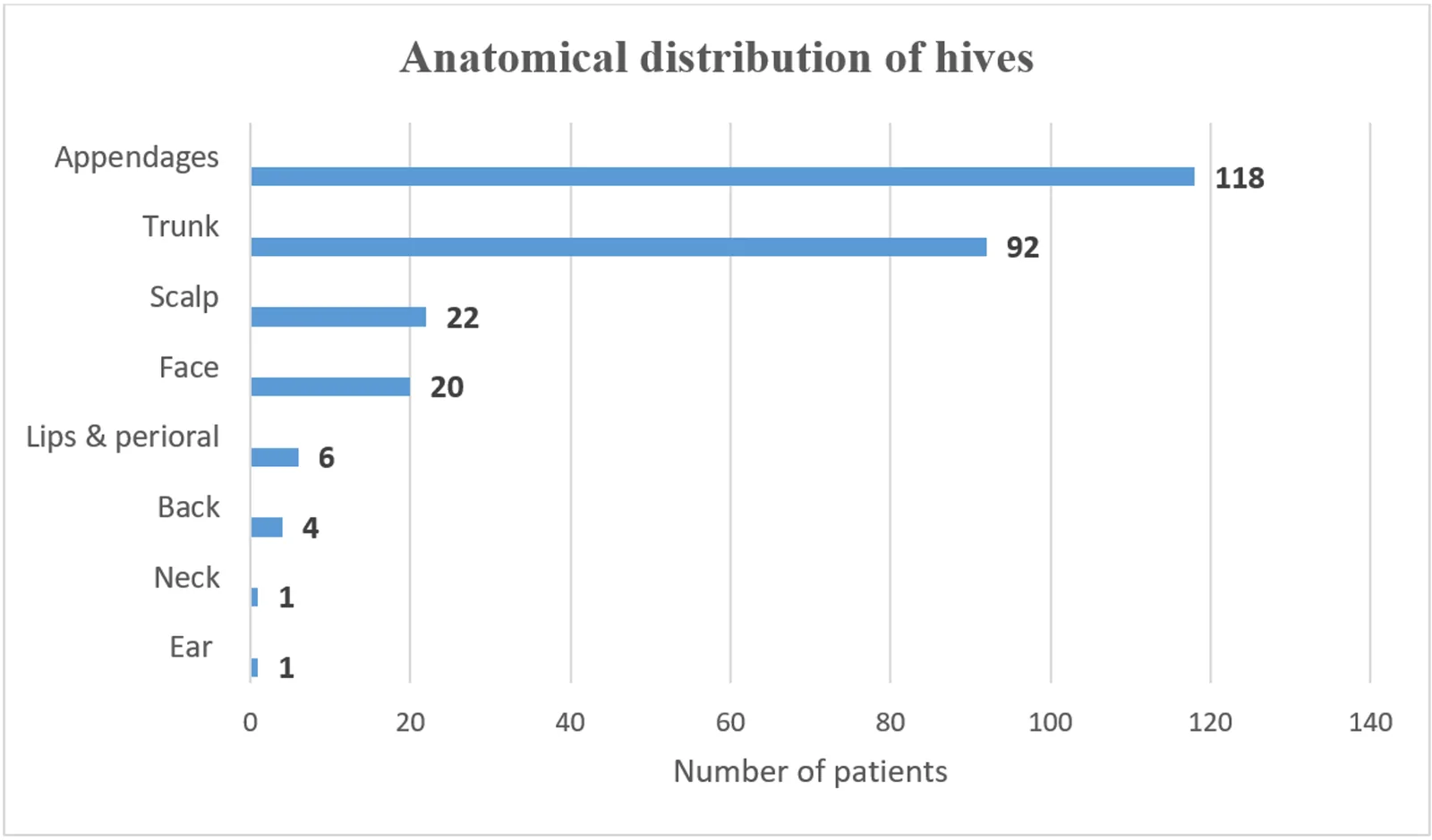
Anatomical distribution of urticarial hives

Patients complained of urticarial hives in several anatomical regions including appendages (118, 95.9%), trunk (92, 74.8%), scalp (22, 17.9%), face (20, 16.3%), and perioral region (6, 4.9%). Other anatomical regions that were involved include back (4, 3.3%), neck (1, 0.8%), and ears (1, 0.8%) (Figure 2).

**Figure 3:**
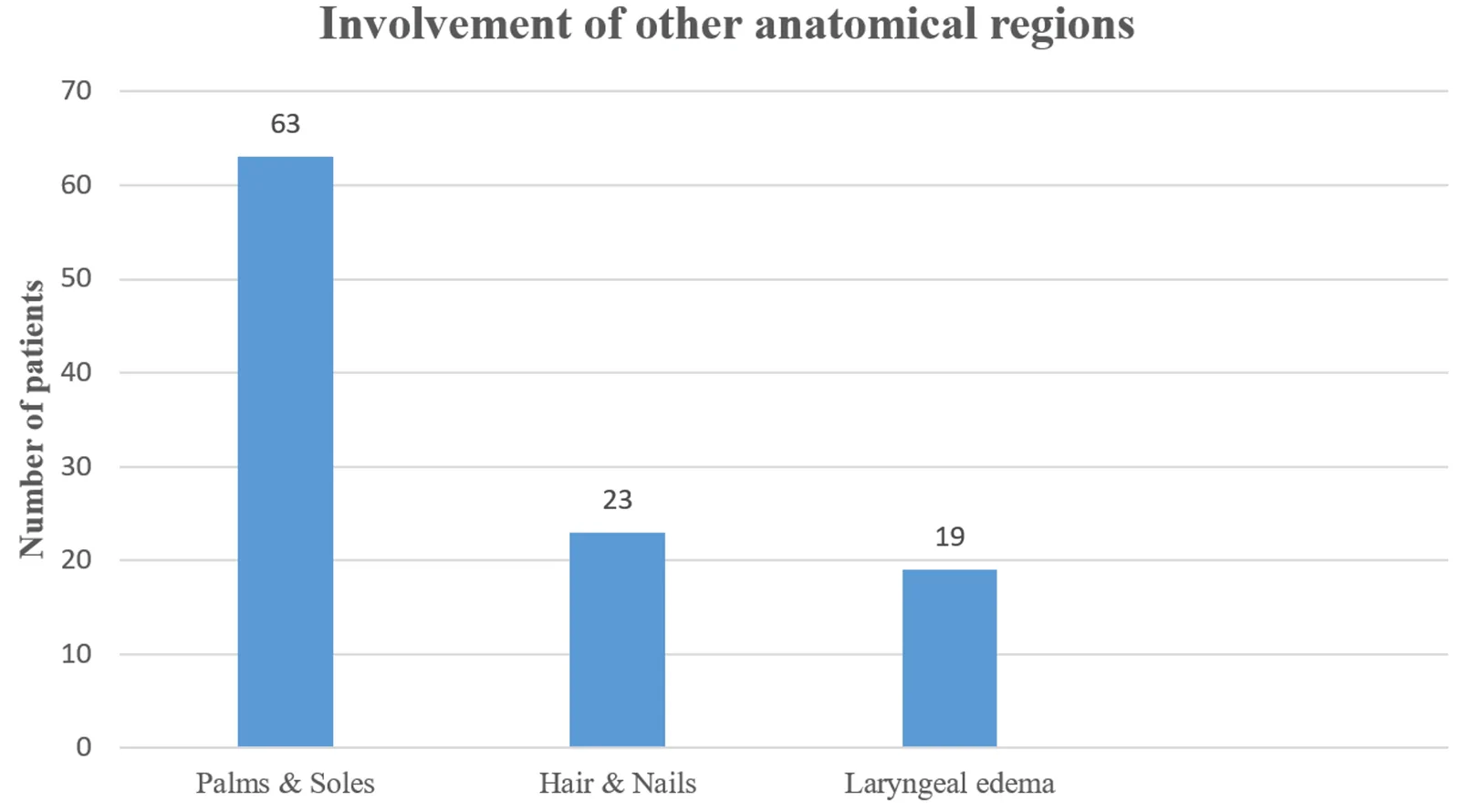
Involvement of other regions

The patients were also asked specific questions to assess involvement of significant regions like palms, soles, hair, nails, and also laryngeal edema, as these can affect the quality of life of the patients. Out of 123 patients, 63 (51.2%) had involvement of palms and soles, 23 (18.7%) had involvement of hair and nails, and 19 (15.4%) had experienced laryngeal edema (Figure 3).

Of the 29 (23.5%) patients with thyroid abnormalities, 26 (89.6%) had involvement of one of the three anatomic loci, namely, palms and soles, hair and nails, and laryngeal edema, compared to 79 of the 94 (84.0%) patients without thyroid abnormalities (Table 3). 

**Table 3:** Involvement of other anatomical regions in patients with thyroid abnormalities

Thyroid abnormality	Palms and soles n (%) p=0.950	Hair and nails n (%) p=0.390	Laryngeal edema n (%) p=1.000	No involvement n (%)	Total n (%)
Present	15 (51.7)	7 (24.1)	4 (13.8)	3 (10.3)	29 (23.5)
Absent	48 (51.1)	16 (17.0)	15 (15.9)	15 (15.9)	94 (76.4)
Total	63 (51.2)	23 (18.6)	19 (15.4)	18 (14.6)	123 (100)

Among patients aged ≥45 years, 34 (51.5%) had involvement of palms and soles compared to 29 (50.9%) among those aged <45 years (Table 4). Hair and nail involvement was seen in 14 (21.2%) patients aged ≥45 years and nine (15.8%) patients aged <45 years (Table 4). Among patients aged ≥45 years, 13 (19.7%) had experienced laryngeal edema compared to 6 (10.5%) among those aged <45 years (Table 4).

**Table 4:** Age-wise distribution of palms and soles involvement, hair and nail involvement, and laryngeal edema

Age category	Total n (%)	Palms and soles involvement p=0.944		Hair and nails involvement p=0.442		Laryngeal edema p=0.161		
		Present n (%)	Absent n (%)	Present n (%)	Absent n (%)	Present n (%)	Absent n (%)
≥45 years	66 (53.7)	34 (51.5)	32 (48.5)	14 (21.2)	52 (78.8)	13 (19.7)	53 (80.3)
< 45 years	57 (46.3)	29 (50.9)	28 (49.1)	9 (15.8)	48 (84.2)	6 (10.5)	51 (89.5)
Total	123 (100)	63 (51.2)	60 (48.8)	23 (18.7)	100 (81.3)	19 (15.4)	104 (84.6)

## Discussion

While there were numerous articles and studies published on the coexistence of CSU and autoimmune conditions, there is limited evidence about screening CSU patients for thyroid disorders like hypothyroidism or hyperthyroidism.

A study done by Kim et al. in 2016 concluded that two (5%) out of 10 patients with autoimmune thyroiditis showed improvement in their urticaria after TSH levels were normalized following treatment with levothyroxine [6].

A case report published by Shahbaz et al. in 2018 supports the positive influence of levothyroxine treatment on resolving symptoms of a patient with CSU and underlying autoimmune thyroiditis [7].

However, a randomized controlled trial conducted by Kiyici et al. in 2010 concluded that there is minimal effect of levothyroxine on CSU in euthyroid patients. It also suggested that an increase in cytokine production could be related to the immunomodulatory effects of TSH suppressive treatment [9].

In this context, the aim of this study was to find out the prevalence of thyroid disorders among patients with CSU. From Table 1, it can be observed that thyroid abnormalities were more frequently noted among patients aged less than 45 years (28.0%) compared to those aged 45 years and above (19.6%), though this difference was not statistically significant (p=0.339). Women were at a higher proportion of the study cohort (69.1%), which is on par with results of other studies like that of Kolkhir et al. [10,11], and this may be explained by the increased incidence of autoimmune thyroid abnormalities in women compared to men [12].

Among those with thyroid abnormalities, hypothyroidism was found to be more prevalent than hyperthyroidism (Figure 1). This could possibly be an implication of the higher prevalence of autoimmune thyroiditis among patients with CSU. According to studies such as that by Kolkhir et al., this autoimmune condition most commonly leads to hypothyroidism rather than hyperthyroidism [10,13].

The relatively narrow IQR suggests a limited variability in thyroid parameter values within the study population.

In Tamil Nadu, reported prevalence of thyroid dysfunction ranges from 12.5% to 32% depending on the population studied, with hospital-based estimates at approximately 22% [14,15]. The 23.5% prevalence observed in our CSU cohort falls within this range, highlighting that thyroid dysfunction is not uncommon in this population and may go undetected without routine screening.

Urticarial hives most commonly involve the trunk and proximal extremities, but lesions may occur anywhere on the body. Areas subjected to pressure or tight-fitting clothing, such as the waistline, axilla, and groin, are also frequently involved [16-19]. In our study, the upper and lower limbs were the most commonly affected sites, as shown in Figure 2. The reason for this finding could be that the hives over the limbs are more visible to the patient than the trunk lesions. Involvement of palms and soles, hair and nails, and laryngeal edema were specifically assessed as these regions significantly influence the quality of life and clinical management. Palms and soles involvement affects activities of daily living, hair and nail changes cause cosmetic distress, and laryngeal edema is a potentially life-threatening complication requiring urgent evaluation.

It is well established that hypothyroidism influences keratinocyte proliferation, hair follicle cycling, and nail matrix activity through direct receptor-mediated effects, manifesting as dry skin, hair thinning, nail brittleness, and mucosal changes including laryngeal myxedema. It can be observed from Table 3 that CSU patients with underlying thyroid abnormalities showed a higher proportion of involvement of other regions, such as hair and nails, compared to euthyroid individuals. However, none of these differences reached statistical significance (palms and soles p=0.950, hair and nails p=0.390, laryngeal edema p=1.000). These findings suggest a possible trend, which requires validation through future studies with larger sample sizes.

A meta-analysis done by Ben-Shoshan et al. concluded that CSU has a significant psychosocial impact on the patients [20-22]. The constant itching can lead to embarrassment and difficulties in social life [23]. Episodes of laryngeal edema in patients with CSU are potentially serious clinical complications and require careful evaluation. Since these three parameters are particularly causing a compromise in the quality of life, it highlights the relevance of testing the thyroid function of patients with CSU. The effect of thyroid status on the distribution of hives was not found to be statistically significant. These findings may achieve statistical significance in studies with larger sample sizes.

Table 4 shows the distribution of clinical features across age groups. While the older age group showed higher rates of urticarial involvement of other regions like palms, soles, hair and nails, and laryngeal edema than the younger age group, none of these differences reached statistical significance (palms and soles p=0.944, hair and nails p=0.442, and laryngeal edema p=0.161). This could possibly be due to age-related variations in immune response [24].

Strengths

The study involved prospective recruitment of patients, which likely minimized, but did not eliminate, recall bias. The inclusion of all eligible consecutive patients improves representativeness of the clinic population and reduces selection bias compared with convenience sampling. A formal sample size calculation was performed prior to data collection, which strengthens the precision of prevalence estimates. Ethical approval was obtained prior to study commencement. Thyroid function was assessed using standardized laboratory testing procedures, which ensures diagnostic accuracy. Age-appropriate laboratory reference ranges were applied for thyroid function classification. Data collection and clinical examination were carried out by a single investigator, which minimizes inter-observer variability.

Limitations

Some analytical tests were not found to be statistically significant because of the relatively small sample size. The absence of a control group is the most significant limitation of this study; hence, comparison of thyroid dysfunction prevalence with the general population and estimation of relative risk could not be done. Causal inference cannot be drawn from this study design, and the reliance on clinical history and examination without biopsy confirmation introduces the possibility of diagnostic misclassification of CSU. The effect of potential confounding factors such as age and gender could not be fully adjusted due to the descriptive cross-sectional study design. As this was a cross-sectional study using consecutive sampling, records of the patients excluded were not maintained. The study was conducted at a single tertiary care center, which limits generalizability. No validated disease severity score was used to assess urticaria severity. The timing of thyroid function testing relative to urticaria treatment was not recorded. Formal pilot testing of the questionnaire was not conducted. Future prospective or case-control studies with larger sample sizes and age-matched controls are recommended. Thyroid autoantibodies, including anti-TPO and anti-thyroglobulin antibodies, were not measured in this study, and therefore autoimmune thyroid disease could not be confirmed. The study did not distinguish between overt and subclinical thyroid dysfunction, which may have implications for the clinical significance of the observed prevalence and restricts comparability with studies that report these categories separately.

## Conclusions

Thyroid dysfunction, predominantly hypothyroidism, was observed in a substantial proportion of patients with CSU in this tertiary care cohort. These findings suggest that thyroid function assessment may be considered in selected CSU patients, particularly where clinical suspicion exists. Larger multicenter controlled studies incorporating thyroid autoantibody testing and disease severity assessment are required to determine whether routine thyroid screening improves clinical outcomes.

## Appendices

**Table 5:** Study questionnaire

General Information	
Date	
Patient ID	
Urticaria Clinic Number	
Name	
Age	
Gender	Male/Female/Other
Occupation	
Contact number	
Residential address	
History	
Duration of urticaria	
Associated comorbidities	Yes/No
Associated ENT concerns	Yes/No
Associated dental issues	Yes/No
Mucosal involvement	Yes/No
History of thyroid-related symptoms	Yes/No
History of thyroid surgery	Yes/No
History of thyroid medication	Yes/No
Clinical examination	
General examination	Normal/Abnormal (specify)
Vitals	
Systemic examination	Normal/Abnormal (specify)
Dermatological Examination	
Distribution of hives	Trunk/Appendages/Both
Duration of concern	
Duration of individual hive	
Mucosal involvement	Yes/No
History of laryngeal edema / respiratory insufficiency	Yes/No
Palms and soles involvement	Yes/No
Hair and nails involvement	Yes/No
Remarks	
